# Beyond the Lab: Cognitive Neuroscience in Real‐World Contexts

**DOI:** 10.1002/wcs.70027

**Published:** 2026-03-10

**Authors:** Stephan P. Kaufhold, Mia Borzello, Federico Rossano, David Kirsh

**Affiliations:** ^1^ Department of Cognitive Science University of California San Diego La Jolla California USA

## Abstract

Cognitive neuroscience has made remarkable advances by conducting rigorously controlled experiments inside the laboratory. However, the generalizability and real‐world relevance of these findings remain limited, in part due to fundamental, often unexamined, assumptions about how cognition operates across species and contexts. In this viewpoint, we critically evaluate three commonly held assumptions underlying current cognitive neuroscience practices: (1) laboratory animals serve as accurate representatives of their wild conspecifics; (2) animal models effectively mirror human cognitive processes; and (3) digital twins provide faithful, functionally equivalent representations of their real‐world analogs. We argue that these assumptions, if left unexamined, risk narrowing our understanding of cognition by excluding the behavioral flexibility, environmental variability, and agency that natural settings afford. We advocate for an expanded notion of ecological validity to include the naturalness of both subjects and environments, and we highlight methodological shifts, such as the use of enriched experimental contexts, mobile neuroimaging, and immersive virtual environments. By reassessing these foundational assumptions, we advocate for an approach to cognitive neuroscience that better reflects the complexity of real‐world behavior, species‐specific cognition, and the environments, physical or virtual, in which cognition is embedded.

## Graphical abstract

Cognitive neuroscience often assumes that using laboratory animals, model species, and digital simulations enables generalizations from lab to wild, from animals to humans, and from virtual to physical. We challenge these assumptions and call for refining ecological validity along three dimensions: subject phenotype, task naturalness, and environmental fidelity.

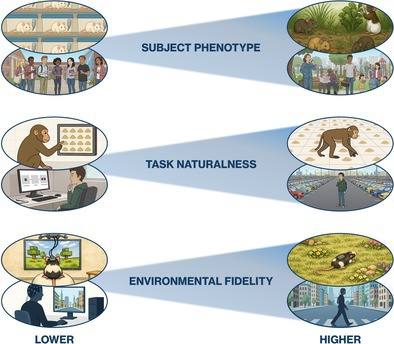

## 1 Introduction

Cognitive neuroscience has made remarkable advances in our understanding of perception, cognition, and behavior through rigorously controlled experiments in the laboratory, which offer precision and causal inference. Methods such as fMRI, EEG, MEG, and single‐unit and multi‐unit recordings enable the field to bridge the gap between neuroanatomy and mental processes. The systematic mapping of neural structures to functions advances our understanding of Marr's (1982) implementation level, that is, how cognitive processes are physically realized in the brain, and Tinbergen's (1963) question of mechanism, which asks about the immediate physiological causes of cognition and behavior. Findings from cognitive neuroscience have yielded both practical and theoretical benefits across many research domains ranging from medicine and neurotechnology (Schalk et al. 2024; Stieglitz 2021; Vázquez‐Guardado et al. 2020) to psychology (Pessoa 2017; Poldrack and Yarkoni 2016) and artificial intelligence (Hassabis et al. 2017). However, the field's focus on the implementational level has been criticized (e.g., Krakauer et al. 2017). Prioritizing experimental rigor and control over complexity comes with trade‐offs: isolating variables in simplified, controlled settings can obscure our understanding of how cognition is embedded in real physical, social, cultural, and developmental contexts (e.g., Rozin 2006). From an evolutionary perspective, perception and cognition evolved to guide behavior and decision‐making in unpredictable, complex environments. Organisms are active agents on the ground with skin in the game that assess local conditions and act autonomously (Tomasello 2022). However, early neuroscientists often treated their subjects as passive stimulus receivers, for example, the pioneering electrophysiological experiments of Hubel and Wiesel (1962) on the visual cortex of cats. Even today, cognitive neuroscience favors constrained experimental environments, both to maintain control over variables and due to constraints inherent to many recording methods. Therefore, experimental methodology can stand in contrast to ecological and enactive perspectives on cognition (e.g., Gibson 1979; Noë 2004). Testing subjects in overly constrained or barren environments risks systematically biasing results by restricting their agency and available affordances. If cognition arises through interactions between agent and environment (Kirsh 1999), then reducing or limiting affordances will constrain the cognitive and behavioral strategies that subjects can deploy. Over time, some methodological choices rooted in convenience and experimental control have become standard practice in the field. While these methodological tensions are broad and multifaceted, we believe three specific assumptions merit particular scrutiny because they form the foundation of how findings generalize across settings and species.

Here, we identify three widely held (often implicit) assumptions in cognitive neuroscience that stem from a lab‐based approach. They enabled much of the field's progress but may also constrain its next steps and generalizability. They are: (1) that laboratory animals are representative of their wild conspecifics; (2) that animal models are appropriate analogs for human cognition; and (3) that digital twins, particularly in VR/AR, are faithful analogs of real‐world environments. Each assumption influences how experiments are designed and interpreted and therefore warrants scrutiny. These assumptions are not just methodologically convenient; they structure how we build experiments, interpret behavior, and draw conclusions. Left unexamined, they risk creating blind spots in our understanding and modeling of minds and brains. Our goal is not to discard these assumptions, but rather to discuss their limits and propose how they should be approached by researchers. We focus on these three in particular, because they reflect a progression of generalizations common across cognitive neuroscience: from lab to wild, from animal to human, and from virtual to physical.

While we considered these three assumptions separately, they commonly co‐occur in practice. Cognitive neuroscience studies typically choose the subjects, tasks, and environments to fit the practical and methodological constraints and norms of the laboratory. This includes using accessible, standard subject populations which are treated as models for broader target populations. For animal subjects, these are often captive or laboratory‐housed cohorts of model species. For human subjects, these are oftentimes WEIRD subjects, that is, subjects of Western, Educated, Industrialized, Rich, Democratic backgrounds, which are not representative of humans in general (Arnett 2008; Henrich et al. 2010). The behavior and neural signals during each task are measured and interpreted within an experimental environment, which is itself a simplification, for example, a test maze in rodents or a constrained computer task in humans. While improving any single dimension can contribute to higher ecological validity, interactions and dependencies should be considered and claims about generalizability should be triangulated (e.g., across studies) through deliberate variation in phenotypes, task naturalness, and environmental fidelity.

We explore here how methodological and technological advances can pave the way for more flexible experimental control outside the laboratory, and for greater richness in the built environments of animals, so that their artificial worlds more closely resemble the wild ones they were shaped by. Finally, we outline a framework for assessing ecological validity across these domains, offering a structured way to balance experimental control with the complexity and variability that characterize cognition in the real world.

## 2 Assumption 1: Laboratory Animals Are Representative of Their Wild Conspecifics

This assumption holds that findings from captive laboratory animal populations generalize to the model species as a whole. The use of laboratory animals in experiments is a cornerstone of cognitive neuroscience and offers researchers a high degree of control. Many studies in cognitive neuroscience rely heavily on model species, such as *C. elegans* , *Drosophila*, zebrafish, mice, rats, and macaques. But do results derived from these species in the lab reliably generalize to their wild conspecifics? If findings fail to generalize even across members of the same species, why trust them as reliable models for human cognition? We question this assumption and discuss how lab animals can differ significantly from their wild counterparts in ways that can systematically bias results.

Data from invertebrates suggest findings *can* generalize: results from laboratory invertebrate populations, for example, *Drosophila*, are equivalent to results from wild ones with no systematic differences in stress tolerance, body‐size traits, and life‐history traits (Maclean et al. 2018). The authors state that “life‐history traits show no evidence for general and directional differences between laboratory and field populations” (p. 540). However, there is a paucity of systematic comparisons between lab animals and their wild counterparts (Alfred and Baldwin 2015), and far less is known about whether such equivalence holds in species with greater behavioral and cognitive flexibility. Laboratory models made substantial contributions to cognitive neuroscience through experimental control and reproducibility. Yet the extent of generalizability they afford remains unclear.

Compared to wild populations, laboratory animals typically exhibit reduced genetic diversity due to domestication, inbreeding, and genetic drift (Kondrakiewicz et al. 2019). Such homogeneity can bias behavioral outcomes, since both genotypic and phenotypic variation are evolutionarily adaptive features of wild populations. Neurocognitive variation is expected to manifest as differences in the distribution of behavior: some individuals may excel at particular tasks while performing worse on others. In natural environments, such behavioral specializations can have adaptive value. In laboratory contexts, animals rarely have the opportunity to display such expertise, yet interindividual differences are evolutionarily meaningful; treating them as noise risks obscuring functionally important cognitive and behavioral specializations (Miller et al. 2022). Researchers should thus analyze data for within‐population variability that may signal such subject types and expand studies to include wild populations where these differences can be observed directly. For example, Snyder‐Mackler et al. (2016) experimentally demonstrated that the immune systems of captive, group‐living macaques adapt to their social status, with low‐ranking individuals exhibiting a shift toward proinflammatory and antibacterial phenotypes and high‐ranking individuals shifted toward antiviral immune phenotypes. This cellular‐level plasticity may be an adaptation against disease risks associated with specific positions within dominance hierarchies. While context‐ and role‐dependent plasticity is oftentimes neglected in laboratory settings, this experiment elegantly demonstrated its presence and relevance within a captive population. Future observational research with wild macaques would be needed to assess whether such findings are generalizable to naturalistic settings.

There is a need for more research using wild populations to look for behavioral, phenotypical or genotypical indicators of subject types to compare with captive populations (Savage and Tremblay 2019). This necessitates a systematic characterization of a variety of subjects' traits. Webster and Rutz (2020) advocated for the STRANGE framework, that is, considering each animal's Social background, Trappability and self‐selection, Rearing history, Acclimation and habituation, Natural changes in responsiveness, Genetic marp, and Experience, to evaluate how representative a study population truly is. Incorporating such standards would clarify the scope of generalizations drawn from laboratory research and support more balanced integrations of control and ecological validity.

The rearing and housing environments of laboratory animals can be additional sources of bias. To understand animal behavior, one should study a species' natural *Umwelt*, that is, its subjective perceptual and ecological niche, defined in terms of time, space, energetic costs and resource distributions (von Uexküll 1934/1957). The dynamic, unconstrained environments wild animals cope with vary in significant ways from the simpler ones facing animals in experimental contexts where their affordances for action are artificially constrained (Pritchett‐Corning 2019). Forcing an animal to make a local choice between going right, left or straight ahead may teach much about the nature of animal wayfinding even in natural environments. But higher‐level goal directed behavior, such as that found in social interaction or non‐navigational problem solving, may require richer contexts than forced choice within a small option set.

Empirical work shows that rearing environments have long‐term effects on cognition and neuroanatomy. For example, rats raised in enriched environments outperform standard‐housed cohorts on memory tasks and exhibit greater adult hippocampal neurogenesis (Ventura et al. 2024). Richer contexts are especially important for animals with higher levels of behavioral flexibility, like passerines, rodents, and primates. Their everyday problems are often orders of magnitude more complex than those faced in experimental contexts. The concept of an animal's *skin in the game* in experiments is often reduced to a behaviorist notion of immediate rewards or punishments. This abstracts too far from what matters to animals in natural contexts, where social dynamics, reproductive goals, and longer‐term strategic considerations shape behavior.

Direct comparisons between wild and captive cohorts further reveal how life history and context shape cognition. *Neophobia*, a reluctance to engage with novelty, is widespread in wild animals but often reduced in captive or habituated individuals. Wild orangutans show markedly lower exploratory behavior toward novel objects compared to zoo‐housed individuals (Forss et al. 2015), and similar patterns occur in vervet monkeys (Forss et al. 2022). These differences likely reflect adaptive wariness in unpredictable environments. Captivity can foster curiosity through routine, safety, and habituation to humans (van Schaik et al. 2016). Domestication can also alter social organization: female laboratory mice form denser social networks and interact more frequently with unrelated individuals than wild‐derived strains (Vogt et al. 2024), and structural brain differences between wild and lab rats likely contribute to such behavioral shifts (Koizumi et al. 2018). Together, these findings show that laboratory populations might express only a narrow subset of species‐typical behavior.

In the wild, animals' behavior results from proximate motivational and learning mechanisms that were shaped by natural selection to increase inclusive fitness. These mechanisms evolved under conditions of predation, competition, and resource uncertainty, which structured the motivational landscape in which animals act. Environments of laboratory animals can shift the motivational ecology by removing or reducing many ecologically relevant pressures and constraints (e.g., predation risks, social competition, scarcity), for example by making resources reliable and removing dangers. Many of the most ecologically relevant selection pressures are precisely those that are difficult (and often unethical) to introduce experimentally. Laboratory researchers should aim to strike a balance between experimental control and ecologically meaningful challenges without introducing ethically problematic dangers, threats or forms of deprivation. Ecological naturalness does not require reproducing predation or scarcity, but preserving functionally meaningful motivations, constraints, and trade‐offs. This aligns with work in animal welfare science arguing that providing animals with opportunities to exercise control by making choices can improve psychological well‐being (Englund and Cronin 2023). Therefore, ecologically meaningful challenges do not require introducing deprivation or dangers as motivators. Instead, they can be implemented by giving captive animals the ability to exert control over their engagement and outcomes (e.g., when, where, and how to participate). The aim, therefore, is not to abandon experimental design in favor of pure observation, but to design experiments and housing conditions that maintain ethical standards while preserving ecologically meaningful contingencies.

New technologies can help accommodate the need for experimental designs that are ecologically natural in both *stimulus richness* and *subject wildness*. This also requires rethinking housing standards for model species. First, to ensure that subjects have more natural living conditions outside of experiments, automation using methods such as RFID or computer vision can enable identification, provisioning, behavior tracking, and health monitoring that improve laboratory animals' health and quality of care. Second, adaptive housing can enrich their built environments thereby raising their complexity or wildness verisimilitude. Third, longitudinal studies of individual animals using big data about the physiology and behavior of animals living in enriched social environments mean that for the first time a whole range of individual differences between animal subjects can become a known variable in animal studies. The same technologies used for monitoring and analyzing behavior within the laboratory can be used to study cognition in animals under more naturalistic settings, such as in semi‐natural habitats or in the wild. Outside the laboratory, researchers have access to a broader range of species, including ones that cannot economically be housed in captivity, providing a reference for behaviors that are species‐typical in naturalistic environments.

We suggest that the notion of ecological validity should be extended to the animals used in observational and experimental contexts. Thus, observing how a domesticated rat behaves in an ecologically natural context does not count as an ecologically valid experiment. What the animal attends to in that natural environment is not likely to be the same as what a conspecific reared in the wild would attend to. The environmental expectations of each and hence their cognition resulting from an active engagement with their environments, that is, their *enactive cognition*, would be different.

Direct comparisons between wild and lab animals remain rare, despite clear evidence that context shapes cognition. This highlights that experiments in controlled environments might model population‐specific forms of cognition that are valid for predicting behavior under those conditions, but not necessarily generalizable to the species as a whole. Tool use and other complex animal behaviors were documented in the wild after decades of lab work missed them (Call 2018). In fact, there are almost no direct comparisons between the cognitive strategies of lab animals and their wild conspecifics (Jacobs and Menzel 2014). The challenge, then, lies in clarifying which level of generalization our findings are meant to support: prediction within a laboratory context or explanation across ecological contexts.

Ultimately, treating animals as passive responders in artificial environments risks severing behavior from the ecological and neural systems that evolved to support it, a methodological convenience that may be systematically obscuring what we set out to study. What needs to be acknowledged is that lab animals only reflect a narrow slice of natural behavior in wild animals. Lab research has focused on stereotyped behaviors in a limited number of species (Miller et al. 2022). Even in well‐studied species, we know little about how brains support dynamic, variable, and infrequent behaviors. Natural behaviors are not statistical noise and rare events may nonetheless play an important role. In the end, there is a need to embrace the distribution of behavior, not filter it out. Behaviors often dismissed as noise or outliers in experimental contexts may in fact be core to how animals engage with the world. If laboratory animals reflect only a constrained slice of their species' behavioral and cognitive repertoire, theories derived from them risk being incomplete or biased. As Gómez‐Marín and Ghazanfar (2014) emphasized, “behavior happens in, and because of, the environment” (p. 7). Laboratory environments restrict not only what animals can do but also what they can perceive, learn, and attend to (Jacobs and Menzel 2014). Even behaviors as seemingly basic as reaction times or exploration tendencies depend on sensory context and prior experience.

Cognition and behavior are contingent on the context in which they are observed. We rarely know the exact socioecological conditions under which they evolved in any given species. Call (2018) argues that testing animals across diverse settings, including laboratory environments that diverge from a species' natural environments, is informative for capturing cognitive flexibility and uncovering latent capacities that might be masked by natural environments. To broaden the diversity and generalizability of findings from laboratory populations, researchers should expand their concept of ecological validity to encompass both the characteristics of the subjects studied and the realism of the context in which they are tested. Key steps to achieve this include (a) enriching laboratory environments to approximate the diversity of natural settings; (b) conducting long‐term, multi‐individual studies that capture variation rather than minimize it (i.e., treating variability as signal rather than noise); and (c) using field‐compatible technologies, such as GPS, bio‐logging, and remote neural recording, to collect behavioral and physiological data in semi‐natural or wild conditions.

In sum, the assumption that laboratory animals accurately represent their wild conspecifics holds under limited conditions. Laboratory animals often differ genetically, behaviorally, and developmentally from wild individuals, leading to systematic biases in the behaviors and neural processes we measure. Recognizing and accounting for these differences is a necessary step toward a cognitive neuroscience that captures the full spectrum and adaptive complexity of minds in their natural worlds.

## 3 Assumption 2: Animals Are Appropriate Models for Human Cognition

As discussed above, translation problems are not exclusively due to experimental task design. They often co‐occur with variation in subject phenotypes and environmental contexts. Therefore, the generalizability of animal models for understanding human cognition discussed below should be interpreted with the other two assumptions in mind. Comparative and translational research are vital parts of cognitive neuroscience for investigating fundamental cognitive and neural processes. Extrapolating from model species to humans has been described as the *backbone of modern biomedical science* (Smulders 2009). This second assumption holds that insights from animal brains and behavior translate directly to human cognition, that is, that studying model species can reveal how human minds work. While we agree that animal models are invaluable, we believe they are at best partial analogss of human cognition due to differences in evolutionary history, neurobiology, and ecological pressures (Akhtar 2015). The oft‐quoted aphorism “what is true for *E. coli* is true for the elephant” is frequently attributed to Jacques Monod (Ball 2023), although it likely originated with Dutch biochemist Albert Kluyver and was popularized through Monod's lectures (Morange 2015). While this aphorism holds weight in molecular biology, its extension to cognitive neuroscience has inspired overly ambitious generalizations. In domains where behavior, cognition, and neuroanatomy vary widely across species, this appealing yet blinkered epistemological framing has shaped research programs for decades. What falls out from this is the need for more refined models and an acknowledgement of the limits of cross‐species translatability.

Mirror neurons are one ongoing debate where questions about cross‐species generalizability are playing out. Discovered in macaques and long interpreted as a mechanistic basis for human social cognition, mirror neurons have been extended to explain uniquely human capacities such as empathy, language, and imitation (Rizzolatti and Craighero 2004). Although mirror‐like activity has been observed in humans through intracranial recordings (e.g., Mukamel et al. 2010; Babiloni et al. 2017), there is still no compelling evidence that the same mechanisms identified in macaques directly support complex human capacities such as empathy, language, or imitation. Recent reviews suggest that mirror neuron areas may primarily contribute to low‐level action processing and movement rather than high‐level sociocognitive abilities (Heyes and Catmur 2022). This shows how enthusiasm for cross‐species continuity can also obscure the real limits of inference. Recognizing those limits allows animal models to inform, rather than define, our understanding of human cognition.

Higher‐order cognitive functions that are distinctively human, such as language, symbolic reasoning, and theory of mind, are unlikely to be fully elucidated through animal models. Rather than treating animals as direct stand‐ins for human cognition, we propose they be best viewed as partial analogss that provide windows into select, phylogenetically conserved processes or analogies under well‐defined constraints. Because humans share a genetic lineage with other animals and possess many homologous structures, it is scientifically reasonable to study processes like learning, memory, perception, and problem‐solving in animals. However, studying animals in ecologically valid contexts can serve as a check against premature generalization. Even outside domains like language or abstract reasoning, animal models offer complementary but incomplete glimpses into human cognition. Like all animals, humans do not perceive the external world directly, but rather as filtered through evolved sensory and cognitive frameworks. This species‐specific filter, or *Umwelt* (von Uexküll 1934/1957), differs meaningfully across species and must be accounted for in comparative designs. Ignoring the *Umwelt* introduces systematic bias. For instance, rodents rely heavily on olfaction, yet are frequently tested using visual or auditory stimuli. Similarly, macaques are often evaluated using human faces rather than conspecifics in social cognition tasks. Such mismatches may mask or distort cognitive abilities, especially in tasks that require explicit representation of goals or rules. Anthropocentric task designs can introduce both sensory and cognitive mismatches across species. This is especially problematic when animal subjects are assumed to explicitly understand task goals or structures. While this assumption more readily holds for linguistic human subjects, it should not be assumed for animals. Notably, designing explicit tasks is even challenging for human subjects, despite there being more ways to communicate goals and instructions (e.g., verbal or written). Piloting and validation experiments are thus commonly required before running explicit tasks with human subjects.

A task is explicit if a subject understands its success conditions; for example, find the shortest path from A to B; choose which items are most similar; point to your starting position; which of the following do you prefer; which of these makes you sad; and so on. The hallmark of explicit tasks is that they require subjects to conceptualize the goal‐state or desired process, which typically depends on linguistic instruction (i.e., words are essential). Animals, by contrast, engage in implicit task performance: they may learn contingencies or associate rewards with behaviors, but do so without access to the metacognitive framing we often take for granted. This disparity has profound implications for cross‐species comparison, especially in domains like social cognition. For instance, Pourtois et al. (2004) found that while the superior temporal cortex in monkeys is involved in processing facial expressions, the same is not true in humans. Instead, the human somatosensory cortex is more involved in processing facial expressions, indicating that superficially similar behaviors can rely on divergent neural architectures.

How, then, can task demands be meaningfully equated across species? This is especially difficult because translating human tasks to animals typically requires training lab animals such as rats and monkeys for weeks, months, or even years. One solution is to design human analogs of well‐established animal paradigms (e.g., virtual mazes) as a means of engaging homologous cognitive processes. Unfortunately, even here, issues of comparability arise. Many human spatial navigation studies now involve using virtual reality (VR) to probe aspects of spatial memory, and these studies often lack the ecologically important features in spatial navigation (e.g., locomotion, translation, and rotation) present in the rodent's physical environment (Taube et al. 2013). When the visual system signals movement but the proprioceptive and vestibular system signal stasis, sensory conflicts arise. The result is reduced realism and a comparability gap that undermines both species' data. Consider the case of theta rhythms: in rodents, ~6–9 Hz theta oscillations are widely accepted as neural markers of navigation and locomotion. However, in humans, VR navigation studies often reveal slower theta rhythms, prompting debate over whether this reflects species difference or methodological artifact. To address this limitation, Bohbot et al. (2017) sought to resolve this inconsistency in an elegant study comparing hippocampal activity from depth electrodes in epilepsy patients during both real‐world and VR navigation. Their results provided the first clear evidence of ~8 Hz theta rhythms in both conditions, suggesting that ecologically rich VR environments may restore some cross‐species comparability.

Technological advances like mobile intracranial EEG (iEEG) are further enabling real‐world cognitive neuroscience by bridging the gap between traditional neuroimaging techniques and real‐world scenarios (Topalovic et al. 2020). Traditionally, brain activity recordings were limited to controlled laboratory settings, restricting the understanding of brain function during natural activities. However, these new tools allow researchers to collect neural data as participants engage in naturalistic tasks, unbound from the laboratory. This breakthrough enables studying brain activity from free‐moving recordings in diverse contexts, providing insights into the neural mechanisms underlying cognition and behavior in ecologically valid conditions. Extended reality (XR) methods, including VR, augmented reality (AR), and mixed reality (MR), can improve stimulus realism and participant agency. AR and MR, in particular, allow participants to interact with real‐world environments overlaid with virtual stimuli, preserving embodied engagement like locomotion, translation, and rotation (e.g., Huffman and Ekstrom 2019; Ruddle and Lessels 2006; Warren et al. 2001) while introducing experimental manipulations. Such XR‐based approaches offer new potential for bridging animal and human paradigms, especially when sensory and motor affordances are matched and aligned more closely with those present in real‐world cognition. Tools like these can narrow the species‐task mismatch by aligning perceptual and motor affordances across contexts.

But even with matched tasks, cognition may differ. Animals and humans often use distinct strategies shaped by their perception, motivation, and neural architecture. Researchers should be cautious to not assume the same mechanisms across species by default. Tailoring tasks so that they align with species‐specific sensory, motivational, and ecological factors can increase both translational value and ethical validity. For instance, rodents typically rely on path integration and olfactory cues, whereas humans favor allocentric strategies involving landmarks and abstract spatial maps (Geva‐Sagiv et al. 2015; Ekstrom 2015). But strategy divergence is not a given. Under specific environmental conditions, both species may converge on shared spatial strategies. For example, Yaski et al. (2011) found that rats navigating urban‐like environments exhibited spatial behavior patterns strikingly similar to those used by humans, suggesting a common underlying structure in certain task ecologies. This finding suggests that task ecology, and not species per se, may drive convergence or divergence in spatial strategies. This reinforces the importance of designing tasks that use ecologically situated methods.

In sum, animal models provide essential but incomplete insights into human cognition, and their utility depends on how well experimental designs accommodate differences in sensory worlds (*Umwelten*), motivation, and evolved strategies. The promise of translation lies not in eliminating these differences, but in designing experiments that respect them. Translation is not about forcing *sameness* but about finding where parallels, whether through homology or analogy, emerge from different bodies and brains.

## 4 Assumption 3: Digital Twins Are Representative of Their Analogue Counterparts

The first two assumptions concern generalization across populations, that is, from laboratory animals to wild conspecifics and from animals to humans. With technological advances enabling virtual task environments, a third assumption is increasingly built into contemporary study designs: that findings obtained in virtual environments will generalize to the corresponding real‐world settings they are intended to simulate.

Digital twins, that is, high‐fidelity virtual simulations of real agents, objects, and environments, are increasingly used in cognitive neuroscience experiments with both humans and animals. Especially XR technologies, such as VR, AR, and MR, are increasingly used as immersive virtual environments for laboratory subjects. It is assumed that if a virtual environment is detailed enough, behavior and neural responses within it will mirror those in corresponding real‐world settings. When executed well, subjects should feel as if they are immersed in a real‐world task. The illusion of reality is, somewhat paradoxically, the point. Successful designs make mediating devices (headsets, screens) perceptually transparent, much like eyeglasses: users do not see them; they see *through* them. This sense of immersion is often cited as the motivation for using VR in cognitive, clinical, and affective neuroscience (Parsons 2015; Kothgassner and Felnhofer 2020). Digital twins promise precise experimental control while preserving the complexity of real environments and enabling continuous, fine‐grained behavioral and physiological logging (Personeni and Savescu 2023). Yet we question this assumption and ask: *How “real” must virtual settings be for findings to generalize*, and *what are the limits of current VR and XR methods in capturing natural behavior?*

Surprisingly few studies have tested whether findings obtained in virtual settings reliably generalize to the physical world and equivalence is often assumed by default. But how immersive must a virtual environment be for cognitive processes to remain analogous? Is a 2D monitor sufficient to evoke spatial memory or motor functions typical of 3D navigation? In practice, many emotion‐recognition or face‐perception studies still rely on two‐dimensional static images rather than dynamic, 3D stimuli (Sonkusare et al. 2019). While such simplifications increase control, they risk distorting or attenuating real‐world cognitive processes. This is especially relevant for cognitive processes that rely on multimodal and spatio‐temporal dynamics. Recent studies show that without depth cues, body movement, or multisensory feedback, behavior in virtual settings does not transfer well to the real world (e.g., Clemenson et al. 2020; McMahan et al. 2016).

Even the most advanced simulations are largely limited to engaging few senses, relying especially on sight and sound with only rudimentary haptic input and little to no olfaction, proprioception, or thermosensation. This can lead to not only incomplete input but disrupted *coherence*. Natural perception is an integrated, multisensory experience rather than an array of separate channels. As Sonkusare et al. (2019) emphasize, our *Umwelt* is inherently multimodal, and any attempt to reproduce it through two channels and a headset will fall short. When sensory cues conflict, as in the *moving train* and *rubber hand* illusion, artificial environments can evoke qualitatively different perceptual states. In VR, study participants may *see* forward motion even though their vestibular system signals no movement. Rather than just engaging in navigation, the brain also has to arbitrate between conflicting cues from different senses. This comes with the risk that an experiment might, at least in part, capture the brain's attempt to reconcile conflicting cues rather than the target cognitive process itself.

These concerns increase when digital twins are designed for nonhuman subjects. Our human intuitions and assumptions about what constitutes salient or relevant features do not necessarily align with an animal's *Umwelt*. While humans are primarily visual, rodents rely heavily on olfaction and whisker‐mediated touch. A visually defined “rodent VR” may fail to engage natural perceptual strategies, or worse, create sensory conflicts that, for example, alter hippocampal coding. Indeed, Aghajan et al. (2015) found that rodent place‐cell activity in virtual environments lacking full sensory dynamics showed degraded spatial selectivity. Such results raise the question of what is actually being measured in animal VR: neural signatures of navigation or artifacts of sensory deprivation. Faithful replication therefore requires attention to species‐specific embodiment and sensory realism, and not just visual fidelity.

Recent advances offer encouraging data that digital twins can approach real‐world fidelity. Studies using open‐space wireless head‐mounted displays, omnidirectional treadmills, and motion tracking (Warren et al. 2001; Ruddle and Lessels 2006; Huffman and Ekstrom 2019) demonstrate that incorporating translational and rotational cues, for example, allowing participants to *walk*, *turn*, and *orient*, greatly enhances spatial realism. In AR and MR, digital elements can be overlaid onto real environments, enabling semi‐naturalistic designs that merge physical and virtual experiences. Such technologies are not limited to studies of navigation but expand to fields such as the neuroscience of architecture, where subtle manipulations of environmental geometry or lighting can be efficiently tested without rebuilding actual structures. Combined with mobile intracranial EEG and other wearable recording tools, XR opens a path toward studying neural dynamics during freely moving, embodied behavior.

Despite this progress, a central question remains: *what level and type of fidelity are necessary for alignment between virtual and real cognition?* XR does not automatically lead to increased ecological validity. The fidelity of digital objects must be empirically tested and conceptually specified. Following Clemenson et al. (2020), researchers should implement veridicality testing, that is, systematically comparing performance and neural signatures across virtual and real tasks, to determine when transfer holds and when it breaks (Hejtmanek et al. 2020). Moreover, digital environments should be designed with the affordances, modalities, and agency that define cognition and behavior in the wild (Gibson 1979; Noë 2004). Too stripped‐down simulations risk reproducing the very shortcomings of earlier laboratory paradigms that treated animals and humans as passive stimulus receivers. Digital twins inherit the assumptions their designers embed, and we risk building elaborate virtual worlds that confirm what we already believed, discovering only what we had the foresight to include.

Ultimately, digital twins' value comes not from exactly copying reality, but from finding the right balance between abstraction and immersion to study cognition. Equivalence should not be assumed by default. Instead, researchers should specify and test the dimensions of ecological alignment (e.g., sensory coherence, embodiment, and species relevance) that are relevant for virtual findings to be generalized to real behavior.

## 5 Conclusion: Toward a Framework for Assessing Ecological Validity

Here, we set out to examine three assumptions that are commonly held in contemporary cognitive neuroscience, namely, (1) that laboratory animals are representative of their wild conspecifics, (2) that animal models provide direct analogss for human cognition, and (3) that digital twins are faithful stand‐ins for their real‐world counterparts. Each assumption reflects an epistemological commitment, the prioritization of control over complexity. Together, they define the conceptual boundaries of what counts as valid evidence in the field. Importantly, the assumptions we discussed often co‐occur in practice. Researchers frequently study laboratory animals (Assumption 1) to draw conclusions about humans (Assumption 2) and increasingly deploy paradigms in virtual environments (Assumption 3). This co‐occurrence means the assumptions can interact, complicating assessments of generalizability and thereby emphasizing that ecological validity is a multidimensional problem.

When these assumptions are held uncritically, they can lead to findings that generalize poorly and might even fail to reflect the cognitive abilities they seek to explain. The methods that enabled past progress, like standardized lab populations, simplified tasks, and virtualized environments, can also constrain what researchers can test and observe. In order to address these limitations, we suggest integrating the strengths of controlled experiments with the complexity of the real‐world ecologies. This can include housing laboratory animals in enriched environments, designing more ethologically grounded tasks, and leveraging emerging technologies, such as mobile EEG, intracranial recordings, and immersive XR platforms, to study cognition emerging from dynamic, embodied contexts (Mobbs et al. 2021).

More broadly, we encourage researchers to be explicit about the trade‐offs they make in the pursuit of precision and to ask what forms of agency, behavior, or cognition may be excluded in the process. Scrutinizing these foundational assumptions can move the field toward a more integrative science of the mind, one that reflects the embeddedness, variability, and situatedness of cognition in the world rather than in isolation from it.

**FIGURE 1:**
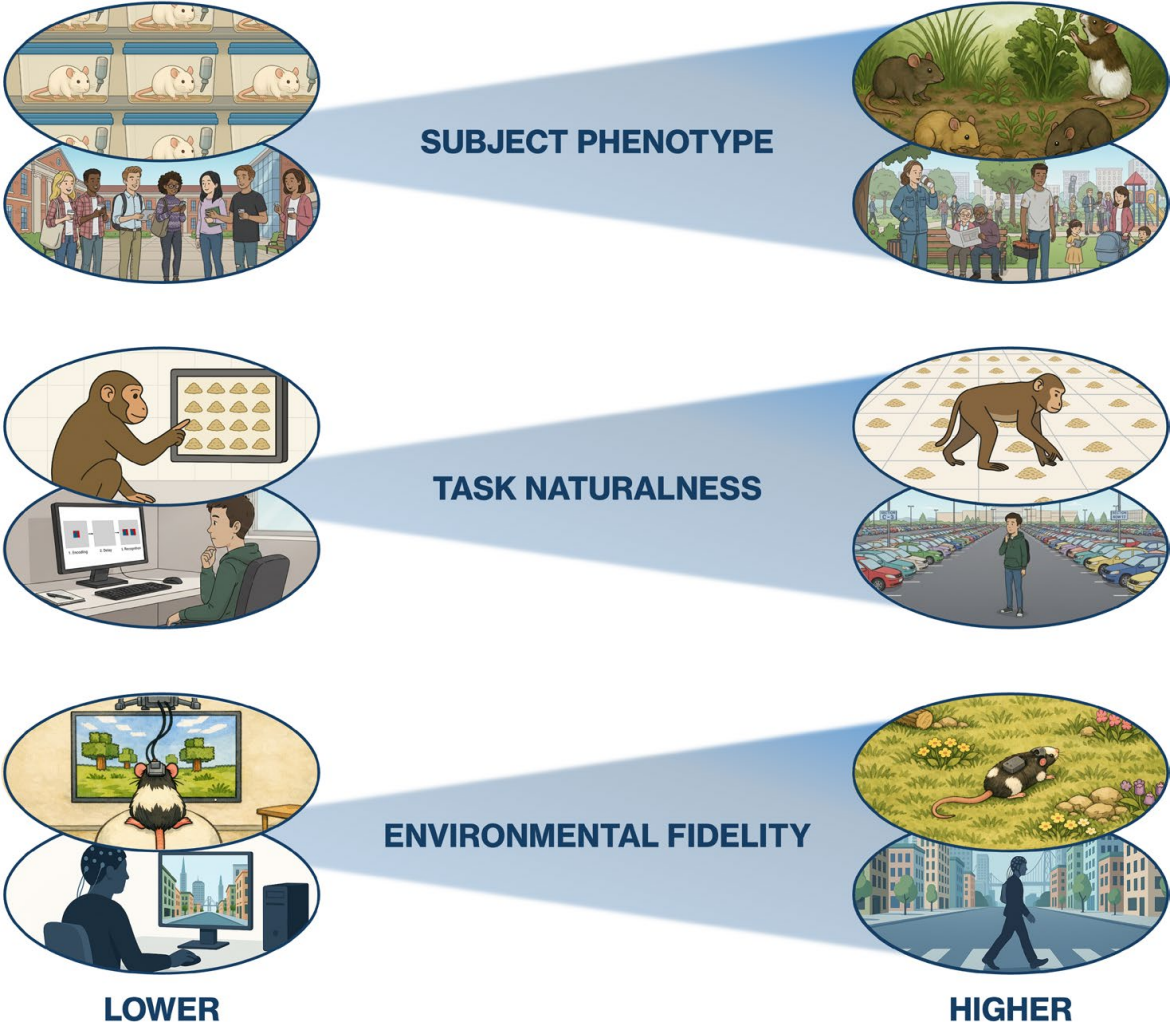
Three graded dimensions of ecological validity in cognitive neuroscience. Ecological validity can vary continuously along subject phenotype, task naturalness, and environmental fidelity, shown here as continua from lower (left) to higher (right) ecological validity to indicate that dimensions are gradual rather than categorical. Each dimension depicts both an animal and a human example. Variation in subject phenotype ranges from single‐housed, laboratory rats and convenience‐sampled college students (lower) to small groups of wild rats foraging together and more diverse human samples (e.g., broader age, occupational, and socio‐economic backgrounds) (higher). Task naturalness ranges from screen‐based tasks (a primate touchscreen foraging task; a human delayed match‐to‐sample task) (lower) to species‐typical, ecologically grounded behaviors (e.g., naturalistic spatial foraging in primates; real‐world spatial memory such as remembering a parking spot in humans, see Means et al. 1995; Postma et al. 2012; da Costa Pinto and Baddeley 1991) (higher). Environmental fidelity ranges from navigation in constrained virtual environments (e.g., rodent VR treadmill paradigms; seated desktop navigation with stationary EEG in humans) (lower) to navigation in physically embodied, real‐world contexts (e.g., semi‐natural enclosures in animals and ambulating through urban environments with mobile EEG in humans) (higher).

If the traditional assumptions of laboratory‐based neuroscience are limiting, what should replace them? We propose that ecological validity should not be treated as a binary property, either “high” or “low”, but as a multidimensional space (Holleman et al. 2020). To make this more explicit, we outline a framework for assessing ecological validity along three complementary axes: the *subject's phenotype*, *task naturalness*, and *environmental fidelity* (see Figure 1). These dimensions together form the basis of what we call ecological alignment, or the degree to which an experimental setup's subjects, tasks, and environments are appropriately matched to the cognitive phenomena under investigation.

### 5.1 Subject Phenotype

How closely does the subject's sensory, motor, and cognitive repertoire resemble that of its wild conspecifics or the population being modeled? This includes consideration of genotype, phenotype, developmental history, and *Umwelt*, that is, the perceptual and subjective world the organism inhabits. Captive or domesticated individuals often differ from wild ones, not just genetically, but also in how they perceive and interact within their environment. These differences shape what counts as a meaningful stimulus or affordance for the specific individual. Researchers should therefore characterize their study population, for example how *STRANGE* (Social background, Trappability, Rearing, Acclimation, Natural responsiveness, Genetic makeup, and Experience; Webster and Rutz 2020) their animal subjects or how WEIRD (Henrich et al. 2010) their human subjects are and consider how such factors influence behavioral and cognitive variability and generalizability.

### 5.2 Task Naturalness

To what extent does the task reflect behaviors or challenges that the subject would encounter in its natural environment? Experimental tasks are not neutral; they define the cognitive problems we assume animals or humans are solving. Tasks should therefore be designed so that their contingencies, affordances, and feedback functions are ecologically meaningful to the species being tested. This encompasses both phylogenetic and ontogenetic ecologies. Promising approaches include using mobile and intracranial EEG to record brain activity during spontaneous behaviors, such as social interaction or navigation, where cognitive demands emerge naturally rather than being artificially imposed (Parodi et al. 2025). Such designs align with 4E perspectives on cognition (embodied, embedded, enacted, extended) that emphasize how reasoning and perception depend on the structure of the task environment itself (Kirsh 1995). Importantly, task naturalness can diminish with overtraining, especially if the learned behavior is arbitrary and not ecologically meaningful to the subject. Thus, the more an animal or subject has to be trained to perform a task within an experiment, the further it may drift from being ecologically valid. This inverse relationship between training and task naturalness should caution experimenters that experimental rigor should be balanced with ecological significance.

### 5.3 Environmental Fidelity

How closely does the physical or digital environment reproduce the perceptual, spatial, and social context in which the behavior under investigation occurs typically? This includes both real and virtual contexts. For example, a digital twin may capture the relevant visual information, but it might miss other meaningful modalities, such as olfaction, touch, or vestibular feedback. The context and affordances that make behavior meaningful can be removed by impoverished environments. Environmental fidelity therefore requires assessing not only visual accuracy but also the *interactional coherence* of perception and action, that is, the degree to which the environment affords the behaviors it seeks to measure. While task naturalness and environmental fidelity are often related in practice, they are conceptually distinct. Task naturalness concerns what the subject is being asked to do (e.g., forage, recall, learn a rule), whereas environmental fidelity concerns where and how that behavior takes place in the environment. For example, a highly natural task (like spatial navigation) may still be performed in a low‐fidelity setting (like in a 2D environment on a touchscreen). Disentangling these variables clarifies how different components of a study contribute, or fail to contribute, to ecological validity.

These three axes can be visualized as coordinates in a multidimensional space of ecological validity. Different experimental paradigms will occupy different regions of this space depending on their goals. A highly controlled neural recording study may deliberately sacrifice environmental fidelity to isolate mechanisms, while a field‐based behavioral study might trade precision for ecological depth. The key is not to maximize all dimensions simultaneously but to make the trade‐offs explicit, that is, to know where an experiment sits in this space and why.

We hope our framework can help with bridging traditional laboratory rigor and ecological realism. Our framework does not mean to replace or discourage controlled experiments, but aims to situate them within a broader continuum that accounts for how cognition is shaped by the interplay of body, task, and environment (Gomez‐Marin et al. 2014). By quantifying where an experiment stands along these dimensions, researchers can better choose their methods with the adaptive functions of cognition in mind and design studies to fill gaps in ecological realism. Both conceptual and technological sophistication can support this. Whether through enriched enclosures, naturalistic field observations, or immersive digital environments, the challenge is to design studies that deliberately consider the ecological and evolutionary grounding of cognition.

Acknowledging the limitations of the three assumptions we discussed here raises the question of how researchers might begin to address them in practice. Researchers can take specific steps to increase the ecological alignment of their study designs while still retaining experimental control. In practice, this will rarely mean that designs can maximize ecological validity across all dimensions at once. Instead, we advocate for adopting incremental changes when feasible, while being explicit about the trade‐offs of decisions regarding subject phenotype, task naturalness, and environmental fidelity. Regarding subject phenotype choices, one practical shift is to characterize rather than minimize variability. Animal researchers should clarify how housing and rearing conditions outside of the experiment deviate from species‐typical (socio‐) ecologies. Similarly, human subject populations should be characterized, and limitations discussed to caution against strong claims of universality. Further, the variation across study subjects should be discussed and analyzed, for example by reporting outliers and characterizing individual strategies. This can contribute to our understanding of the robustness of effects in relation to specific phenotypes and ecologies. Similarly, tasks and environments should not be evaluated by performance metrics alone but also by ecological plausibility. Specific behaviors and associations that subjects must learn for the task should be ecologically meaningful or functional rather than arbitrary. Instead of arbitrary shaping, training subjects should primarily help them understand the affordances and contingencies of the experimental design. When possible, tasks should include decision points and behavioral alternatives to increase subjects' choice and agency. Finally, environmental fidelity can be improved by giving subjects additional affordances and behavioral alternatives that introduce opportunity costs, effort, or uncertainty (rather than deprivation or danger). Researchers should aim to embed experiments in physical and social environments that are meaningful to subjects and when virtual environments are used, they should recreate those affordances with high fidelity. Acknowledging that each experiment has limitations and trade‐offs, researchers should communicate which dimensions of ecological validity were prioritized and which were constrained in any given design. By shifting from control versus complexity to control *with* complexity, cognitive neuroscience can move beyond the lab and toward a science of cognition that is as dynamic and context‐sensitive as the phenomena it seeks to explain (Rosati et al. 2022; Gomez‐Marin and Ghazanfar 2019).

## Author Contributions

**Stephan P. Kaufhold:** conceptualization, writing – review and editing, writing – original draft, visualization, project administration. **Mia Borzello:** conceptualization, writing – review and editing, writing – original draft, visualization, project administration. **Federico Rossano:** writing – review and editing, supervision. **David Kirsh:** conceptualization, writing – review and editing, writing – original draft, supervision.

## Funding

The authors have nothing to report.

## Conflicts of Interest

The authors declare no conflicts of interest.

## Data Availability

Data sharing not applicable to this article as no datasets were generated or analysed during the current study.
